# O02 - Exercise-induced bronchoconstriction in young athletes

**DOI:** 10.1186/2045-7022-4-S1-O2

**Published:** 2014-03-14

**Authors:** Sven Seys, Gudrun Marijsse, Ellen Dilissen, Thierry Troosters, Jan Ceuppens, Koen Peers, Lieven Dupont, Dominique Bullens

**Affiliations:** 1 Catholic University of Leuven, Leuven, Belgium

## Introduction

Exercise induced bronchoconstriction (EIB) is more prevalent in elite athletes compared to controls. It is however unclear how many young athletes suffer from EIB.

## Methods

Football players (n=24), basketball players (n=15), swimmers (n=12) were recruited at the elite sport high school (12-14 years old) in Leuven (Belgium). Age-matched controls (n=7) were recruited among children performing sports at a recreational level. Eucapnic voluntary hyperventilation test was used to assess EIB according to previous standards. Subjects breathed a gas mixture (5% CO_2_, 21% O_2_ and 74% N_2_) at a target rate of 85% of their maximal voluntary ventilation (MVV) per minute (assessed before the EVH test) for 6 minutes. Spirometry was performed at 1, 5, 10 and 15 min after the EVH challenge. EVH test was considered positive if the fall in FEV_1_ ≥10%. Allergy for house dust mite, grass pollen, tree pollen, weeds, dog and moulds was assessed by skin prick test (considered positive if at least one SPT was positive).

## Results

FVC (L) was significantly higher in swimmers compared to controls (p<0.05). EIB (fall in FEV_1_ ≥10% at EVH test) was diagnosed in 4 out of 12 swimmers, 3 out of 20 football players, 1 out of 11 basketball players and 1 out of 7 control individuals. Only 1 of these individuals (swimmer) had pre-existing asthma. Maximal fall in FEV_1_ (%) was significantly higher in swimmers (mean: -8.8%) compared to football players (mean: -6.1%), basketball players (mean: -1.0%) and controls (mean: -3.6%) (p=0.027). Allergy was equally distributed among four groups: 7 out of 24 football players, 1 out of 7 controls, 5 out of 11 basketball players, 3 out of 11 swimmers (p=0.94).

## Conclusion

Swimmers had highest prevalence of EIB. Maximal fall in FEV_1_ was significantly higher in swimmers compared to other athletes and controls despite higher FVC levels. Competitive swimmers are exposed to both intense exercise and airborne trichloramine in contrast to other athletes (only intense exercise) and controls. This might explain why airway hyperreactivity is more common in swimmers compared to other athletes.

